# Comparison of Outcomes After Transcatheter Aortic Valve Replacement vs Surgical Aortic Valve Replacement Among Patients With Aortic Stenosis at Low Operative Risk

**DOI:** 10.1001/jamanetworkopen.2019.5742

**Published:** 2019-06-14

**Authors:** Marko P. O. Virtanen, Markku Eskola, Maina P. Jalava, Annastiina Husso, Teemu Laakso, Matti Niemelä, Tuomas Ahvenvaara, Tuomas Tauriainen, Pasi Maaranen, Eeva-Maija Kinnunen, Sebastian Dahlbacka, Jussi Jaakkola, Tuija Vasankari, Juhani Airaksinen, Vesa Anttila, Stefano Rosato, Paola D’Errigo, Mikko Savontaus, Tatu Juvonen, Mika Laine, Timo Mäkikallio, Antti Valtola, Peter Raivio, Fausto Biancari

**Affiliations:** 1 Heart Hospital, Tampere University Hospital, Faculty of Medicine and Health Technology, Tampere, Finland; 2 Heart Center, Turku University Hospital, Turku, Finland; 3 Heart Center, Kuopio University Hospital, Kuopio, Finland; 4 Heart Center, Helsinki University Hospital, Helsinki, Finland; 5 Department of Internal Medicine, Oulu University Hospital, Oulu, Finland; 6 Department of Surgery, Oulu University Hospital, University of Oulu, Oulu, Finland; 7 National Centre of Global Health, Istituto Superiore di Sanità, Rome, Italy; 8 Department of Surgery, University of Turku, Turku, Finland

## Abstract

**Importance:**

Transcatheter aortic valve replacement (TAVR) has been shown to be a valid alternative to surgical aortic valve replacement (SAVR) in patients at high operative risk with severe aortic stenosis (AS). However, the evidence of the benefits and harms of TAVR in patients at low operative risk is still scarce.

**Objective:**

To compare the short-term and midterm outcomes after TAVR and SAVR in low-risk patients with AS.

**Design, Setting, and Participants:**

This retrospective comparative effectiveness cohort study used data from the Nationwide Finnish Registry of Transcatheter and Surgical Aortic Valve Replacement for Aortic Valve Stenosis of patients at low operative risk who underwent TAVR or SAVR with a bioprosthesis for severe AS from January 1, 2008, to November 30, 2017. Low operative risk was defined as a Society of Thoracic Surgeons Predicted Risk of Mortality score less than 3% without other comorbidities of clinical relevance. One-to-one propensity score matching was performed to adjust for baseline covariates between the TAVR and SAVR cohorts.

**Exposures:**

Primary TAVR or SAVR with a bioprosthesis for AS with or without associated coronary revascularization.

**Main Outcomes and Measures:**

The primary outcomes were 30-day and 3-year survival.

**Results:**

Overall, 2841 patients (mean [SD] age, 74.0 [6.2] years; 1560 [54.9%] men) fulfilled the inclusion criteria and were included in the analysis; TAVR was performed in 325 patients and SAVR in 2516 patients. Propensity score matching produced 304 pairs with similar baseline characteristics. Third-generation devices were used in 263 patients (86.5%) who underwent TAVR. Among these matched pairs, 30-day mortality was 1.3% after TAVR and 3.6% after SAVR (*P* = .12). Three-year survival was similar in the study cohorts (TAVR, 85.7%; SAVR, 87.7%; *P* = .45). Interaction tests found no differences in terms of 3-year survival between the study cohorts in patients younger than vs older than 80 years or in patients who received recent aortic valve prostheses vs those who did not.

**Conclusions and Relevance:**

Transcatheter aortic valve replacement using mostly third-generation devices achieved similar short- and mid-term survival compared with SAVR in low-risk patients. Further studies are needed to assess the long-term durability of TAVR prostheses before extending their use to low-risk patients.

## Key Points

**Question:**

Does transcatheter aortic valve replacement achieve similar results compared with surgical aortic valve replacement in patients at low operative risk with severe aortic stenosis?

**Findings:**

In this comparative effectiveness cohort study of 2841 low-risk patients with aortic stenosis from Finalnd, propensity score–matching analysis showed similar 30-day and 3-year survival after transcatheter aortic valve replacement and surgical aortic valve replacement.

**Meaning:**

Patients with severe aortic stenosis at low operative risk may be offered transcatheter aortic valve replacement instead of surgical aortic valve replacement.

## Teaser

This comparative effectiveness cohort study examines 30-day and 3-year survival among Finnish patients with aortic stenosis at low operative risk who underwent transcatheter aortic valve replacement compared with surgical aortic valve replacement.

## Introduction

The development of transcatheter aortic valve replacement (TAVR) has made the treatment of severe aortic stenosis (AS) feasible with similar short- and mid-term outcomes compared with surgical aortic valve replacement (SAVR) in patients with high^1,2,3^ or intermediate^4,5,6^ operative risk. Clinical practice has recently turned toward treating even low-risk patients with TAVR, and 3 recent randomized clinical trials reported favorable short-term results with TAVR in these patients.^7,8,9^ The Evolut Low Risk Trial^7^ documented a 2-year mortality of 4.5% after either TAVR or SAVR. The PARTNER 3 Trial^8^ reported a 1-year mortality of 1.0% after TAVR and 2.5% after SAVR. The Nordic Aortic Valve Intervention Trial (NOTION)^9,10^ randomized patients to receive TAVR or SAVR, and 82% of the patients were at low risk for surgical operations, ie, Society of Thoracic Surgeons Predicted Risk of Mortality (STS-PROM) score less than 4%. Similar outcomes were achieved in both TAVR and SAVR treatment arms at 6 years.^9,10^ A 2018 study^11^ showed that transfemoral TAVR using mainly a third-generation balloon-expandable TAVR device was associated with no deaths at 30 days compared with 1.7% in a historical, propensity-matched SAVR cohort. However, the long-term durability of TAVR prostheses in low-risk populations is questionable based on registry data, to our knowledge.^12^ This leaves uncertainty whether TAVR is an acceptable treatment for low-risk patients. The aim of this study was to compare the short-term and midterm survival of low-risk patients treated with TAVR and SAVR in a nationwide study.

## Methods

### Study Design and Participants

The Nationwide Finnish Registry of Transcatheter and Surgical Aortic Valve Replacement for Aortic Valve Stenosis (FinnValve registry) is a study (ClinicalTrials.gov identifier, NCT03385915) that includes retrospectively collected data from consecutive and unselected patients treated with TAVR or SAVR with bioprostheses for AS from January 1, 2008, to November 30, 2017, at all 5 university hospitals in Finland (Helsinki University Hospital, Helsinki, Finland; Kuopio University Hospital, Kuopio, Finland; Oulu University Hospital, Oulu, Finland; Tampere University Hospital, Tampere, Finland; and Turku University Hospital, Turku, Finland). The study followed the Strengthening the Reporting of Observational Studies in Epidemiology (STROBE) reporting guideline. The study protocol was approved by the institutional review boards of all participating centers. Informed consent was waived because of the retrospective nature of this study. The inclusion criteria for study entry were age older than 18 years, previous primary aortic valve procedure with a bioprosthesis for AS with or without associated regurgitation, and TAVR or SAVR with or without associated coronary revascularization. The exclusion criteria were any prior TAVR or surgical intervention on the aortic valve; a concomitant major procedure on the mitral valve, tricuspid valve, or ascending aorta; active endocarditis; or any procedure for isolated aortic valve regurgitation. The operative risk of patients was stratified according to STS-PROM^13^ and updated European System for Cardiac Operative Risk Evaluation (EuroSCORE II)^14^ scores. Exclusion criteria included having an STS-PROM score of 3% or higher, undergoing an urgent or emergency procedure, having previously undergone a cardiac surgical operation, being older than 85 years, undergoing chronic dialysis, having a functioning kidney transplant, having severe frailty, having an active malignancy, having had a recent episode of acute heart failure, having a porcelain aorta, being treated with oxygen therapy, having a left ventricular ejection fraction of 30% or below, having a severe mitral valve regurgitation, or not having transfemoral access for TAVR (eFigure in the Supplement).

Data were retrospectively collected in a dedicated electronic case report form by cardiologists, cardiac surgeons, and trained research nurses from December 1, 2017, to July 31, 2018, and underwent robust checking of its completeness and quality. Data on mortality were retrieved from the Finnish national registry Statistics Finland. Follow-up was considered complete for all patients, but follow-up was truncated at hospital discharge for those not residing in Finland. Analyses were conducted October 29, 2018, through November 7, 2018.

### Baseline Risk Factors

Baseline variables were defined according to the EuroSCORE II criteria.^14^ Severe frailty was defined as Geriatric Status Scale^15^ grades 2 and 3. Coronary artery disease (CAD) was defined as any stenosis of 50% or more of the main coronary branches. Recent acute heart failure was defined as new-onset or worsening of heart failure requiring hospital admission within 60 days prior to intervention.

### Outcome Measures

The primary outcomes were 30-day and 3-year survival. The secondary outcomes were stroke, blood transfusion, bleeding, resternotomy for bleeding, paravalvular regurgitation, new permanent pacemaker implantation, acute kidney injury, renal replacement therapy, conversion to cardiac surgical procedure, coronary artery occlusion, aortic dissection or rupture, major vascular complication, atrial fibrillation, postoperative length of stay in the hospital where the procedure was performed, and repeated aortic valve replacement.

Stroke and major vascular complications were defined according to the Valvular Academic Research Consortium-2 criteria.^16^ Major bleeding was defined as European Multicenter Study on Coronary Artery Bypass Grafting bleeding grades 2 or 3, ie, transfusion of more than 4 units of red blood cells or resternotomy for excessive bleeding.^17^ In this study, the Valvular Academic Research Consortium-2 definition of major and life-threatening bleeding was not applied because, unlike patients undergoing TAVR, a significant decrease of hemoglobin level is observed in most patients undergoing SAVR, and this does not always reflect a condition of major perioperative blood loss. Acute kidney injury was defined according to the Kidney Disease: Improving Global Outcomes classification criteria,^18^ ie, stage 1 is an increase in serum creatinine levels of at least 1.5-fold the baseline level or a serum creatinine level increase of at least 0.3 mg/dL (to convert to micrograms per liter, multiply by 88.4); stage 2 is an increase in serum creatinine level 2.0- to 2.9-fold the baseline; and stage 3 is defined as an increase in serum creatinine concentration at least 3-fold the baseline level or a serum creatinine concentration increase at least 4.0 mg/dL during the hospital stay or de novo renal replacement therapy during the hospital stay.

### Statistical Analysis

Statistical analysis was performed using SAS statistical software version 9.2 (SAS Institute) and SPSS statistical software version 25.0 (IBM). Continuous variables are reported as means and SDs as well as median and interquartile ranges, while categorical variables are reported as counts and percentages. Mann-Whitney *U* test, Fisher exact test, and χ^2^ test were used for univariate analysis in the unmatched population. Missing data were not replaced. A propensity score was estimated using a nonparsimonious logistic regression model, including age, sex, body mass index, hemoglobin, estimated glomerular filtration rate, diabetes, stroke, transient ischemic attack, pulmonary disease, extracardiac arteriopathy, New York Heart Association class 4 symptoms, left ventricular ejection fraction of 50% or less, atrial fibrillation, pulmonary artery pressure, recent myocardial infarction, CAD, left main coronary stenosis, number of diseased coronary arteries, moderate mitral valve regurgitation, and prior pacemaker as covariates. One-to-one propensity score matching was performed using the nearest-neighbor method and a caliper width of 0.2 of the SD of the logit of the propensity score. Furthermore, 3 different propensity score–matching analyses were performed addressing exact matching of patients older than 80 years, with CAD, and with selected valve prostheses (ie, third-generation TAVR prostheses and their variants (ie, EvolutR, Sapien 3, ACURATE neo, and Lotus) and selected SAVR prostheses and their variants with proven durability (ie, Trifecta, Perimount).^19^ These matched data sets were used for interaction tests analyses. To evaluate the balance between the matched groups, the *t* test for paired samples for continuous variables, the McNemar test for dichotomous variables, and the analysis of the standardized differences after matching were used. Standardized differences less than 0.10 were considered an acceptable imbalance between the treatment groups. Early outcomes in the propensity score–matched cohorts were evaluated using the *t* test for paired samples for continuous variables and the McNemar test for dichotomous variables. These tests were used to evaluate any difference in the adverse events of propensity score–matched pairs. Differences in the long-term survival of matched pairs were evaluated using the Kaplan-Meier method with the Klein-Moeschberger stratified log-rank test. *P* values were 2-tailed, and a *P* value less than .10 was considered statistically significant for interaction tests of matched cohorts. A *P* value less than .05 was considered statistically significant for all the other tests.

## Results

The FinnValve registry includes data from 6463 patients who underwent primary TAVR or SAVR with a bioprosthesis for severe AS. Of these, 2841 patients (mean [SD] age, 74.0 [6.2] years; 1560 [54.9%] men) fulfilled the inclusion criteria and were included in analysis (eFigure in the Supplement). Surgical aortic valve replacement was performed in 2516 patients, and TAVR was performed in 325 patients. A significant interinstitutional difference in the prevalence of low-risk patients undergoing TAVR was observed (Helsinki, 102 patients [11.0%]; Kuopio, 75 patients [18.7%]; Oulu, 74 patients [13.2%]; Tampere, 20 patients [3.4%]; Turku, 54 patients [14.8%]; *P* < .001). The mean (SD) follow-up of this series was 4.0 (2.7) years (TAVR cohort, 1.7 [1.4] years; SAVR cohort, 4.3 [2.7] years).

### Characteristics and Outcomes of the Unmatched Cohorts

The baseline characteristics of the low-risk patients in the unmatched TAVR and SAVR groups are shown in Table 1. Patients who underwent SAVR were younger, more often men, and had lower STS-PROM and EuroSCORE II risk scores compared with patients in the TAVR cohort (Table 1). Before matching, the prevalence of previous stroke, pulmonary disease, peripheral arteriopathy, atrial fibrillation, and mitral regurgitation was higher in the TAVR cohort compared with the SAVR cohort. Patients in the SAVR cohort had a higher prevalence of CAD and more often underwent concomitant revascularization.

**Table 1.** Characteristics of Unmatched Patients With Low Operative Risk Undergoing Transcatheter or Surgical Aortic Valve Replacement^a^

Characteristic	No. (%)		Standardized Difference (95% CI)	*P* Value
	TAVR (n = 325)	SAVR (n = 2516)		
Age, y				
Mean (SD)	78.1 (6.0)	73.4 (6.0)	0.77 (0.66 to 0.77)	<.001
Median (IQR)	80.0 (75.7-82.1)	74.0 (69.9-77.7)		
Men	153 (47.1)	1407 (55.9)	0.18 (0.06 to 0.29)	.003
Body mass index^b^				
Mean (SD)	28.6 (5.1)	28.0 (4.7)	0.12 (0.01 to 0.24)	.05
Median (IQR)	27.9 (24.7-31.9)	27.4 (24.6-30.8)		
Hemoglobin, g/dL^c^				
Mean (SD)	13.0 (1.5)	13.4 (1.3)	0.38 (0.27 to 0.50)	<.001
Median (IQR)	13.1 (12.1-14.0)	13.6 (12.7-14.5)		
eGFR, mL/min/1.73 m^2^^c^				
Mean (SD)	76 (21)	80 (20)	0.20 (0.09 to 0.32)	<.001
Median (IQR)	73 (62-88)	79 (66-92)		
Diabetes	75 (23.1)	555 (22.1)	0.02 (−0.09 to 0.14)	.68
Stroke	29 (8.9)	127 (5.0)	0.15 (0.04 to 0.27)	.004
Transient ischemic attack	22 (6.8)	107 (4.3)	0.11 (−0.01 to 0.23)	.04
Pulmonary disease	60 (18.5)	275 (10.9)	0.21 (0.10 to 0.33)	<.001
Extracardiac arteriopathy	41 (12.6)	207 (8.2)	0.14 (0.03 to 0.26)	.008
LVEF ≤50%	47 (14.5)	298 (11.8)	0.08 (−0.04 to 0.19)	.18
Atrial fibrillation	118 (36.3)	457 (18.2)	0.42 (0.30 to 0.53)	<.001
NYHA class 4	7 (2.2)	20 (0.8)	0.11 (−0.002 to 0.23)	.02
SPAP, mm Hg				
31-55	109 (33.5)	799 (31.8)	0.12 (0 to 0.23)	.11
>55	18 (5.5)	86 (3.4)		
Moderate mitral regurgitation	25 (7.7)	77 (3.1)	0.22 (0.10 to 0.33)	<.001
Recent myocardial infarction	4 (1.2)	20 (0.8)	0.04 (−0.08 to 0.16)	.42
Coronary artery disease	60 (18.5)	877 (34.9)	0.38 (0.26 to 0.49)	<.001
Left main coronary stenosis	2 (0.6)	76 (3.0)	0.18 (0.07 to 0.30)	.01
No. of diseased vessels				
Mean (SD)	0.2 (0.5)	0.6 (0.9)	0.47 (0.36 to 0.59)	<.001
Median (IQR)	0 (0)	0 (0-1.0)		
Prior PCI	65 (20.0)	200 (7.9)	0.35 (0.24 to 0.47)	<.001
Permanent pacemaker	24 (7.4)	90 (3.6)	0.17 (0.05 to 0.28)	.001
Planned concomitant PCI or CABG	9 (2.8)	812 (32.3)	0.84 (0.72 to 0.96)	<.001
EuroSCORE II, %				
Mean (SD)	2.6 (1.5)	2.1 (1.1)	0.42 (0.31 to 0.54)	<.001
Median (IQR)	2.2 (1.7-3.2)	1.8 (1.3-2.6)		
STS-PROM score, %				
Mean (SD)	2.1 (0.5)	1.8 (0.6)	0.63 (0.51 to 0.74)	<.001
Median (IQR)	2.2 (1.8-2.5)	1.7 (1.3-2.2)		

Abbreviations: CABG, coronary artery bypass grafting; eGFR, estimated glomerular filtration rate; EuroSCORE II, updated European System for Cardiac Operative Risk Evaluation; IQR, interquartile range; LVEF, left ventricular ejection fraction; NYHA, New York Heart Association functional classification; PCI, percutaneous coronary intervention; SAVR, surgical aortic valve replacement; SPAP, systolic pulmonary artery pressure; STS-PROM, Society of Thoracic Surgeons Predicted Risk of Mortality; TAVR, transcatheter aortic valve replacement.

SI conversion factor: To convert hemoglobin to grams per liter, multiply by 10.

^a^ Clinical variables are according to the EuroSCORE II definition criteria.

^b^ Calculated as weight in kilograms divided by height in meters squared.

^c^ Calculated using the original Modification of Diet in Renal Disease equation.

In unmatched cohorts, 4 patients (1.2%) in the TAVR cohort and 50 patients (2.0%) in the SAVR cohort died within 30 days (*P* = .52). Early outcomes of the unadjusted cohorts are presented in Table 2. Three-year survival was lower for the TAVR cohort (85.5%) compared with the SAVR cohort (92.0%), but the difference was not statistically significant (*P* = .20).

**Table 2.** Early Outcomes of Unmatched Low-Risk Patients Undergoing TAVR or SAVR

Outcome	No. (%)		*P* Value
	TAVR (n = 325)	SAVR (n = 2516)	
Deaths within 30 d	4 (1.2)	50 (2.0)	.52
Conversion to cardiac surgery	3 (0.9)	NA	NA
Coronary ostium occlusion	2 (0.6)	9 (0.4)	.36
Aortic dissection/rupture	2 (0.6)	19 (0.8)	>.99
Major vascular complication	28 (8.6)	36 (1.4)	<.001
Stroke	6 (1.8)	76 (3.0)	.23
RBC transfusion, units			
Mean (SD)	0.4 (1.2)	2.3 (3.3)	<.001
Median (IQR)	0 (0)	2.0 (0-3.0)	
RBC transfusion >4 units	8 (2.5)	353 (14.2)	<.001
Resternotomy for bleeding	1 (0.3)	190 (7.6)	<.001
E-CABG bleeding grades 2-3	8 (2.5)	418 (16.8)	<.001
KDIGO acute kidney injury grades 2-3	6 (1.9)	111 (4.4)	.03
Renal replacement therapy	1 (0.3)	39 (1.6)	.08
Paravalvular regurgitation			
Mild	48 (14.8)	135 (5.4)	<.001
Moderate	9 (2.8)	10 (0.4)	
Severe	0	4 (0.2)	
Atrial fibrillation	100 (30.8)	1330 (52.9)	<.001
Permanent pacemaker	35 (10.8)	98 (3.9)	<.001
Hospital stay, d			
Mean (SD)	4.1 (3.2)	7.7 (5.7)	<.001
Median (IQR)	4.0 (2.0-5.0)	7.0 (5.0-8.0)	

Abbreviations: E-CABG, European Coronary Artery Bypass Grafting registry; IQR, interquartile range; KDIGO, Kidney Disease: Improving Global Outcomes; NA, not applicable; RBC, red blood cells; SAVR, surgical aortic valve replacement; TAVR, transcatheter aortic valve replacement.

### Characteristics and Outcomes of the Propensity Score–Matched Cohorts

Propensity score matching produced 304 pairs of patients with similar baseline characteristics (Table 3). The standardized differences between groups were less than the prespecified margin indicating good balance of covariates. The rate of planned concomitant revascularization was lower in the TAVR group compared with the SAVR group (6 patients [2.0%] vs 49 patients [16.1%]; *P* < .001). The prevalence of CAD (57 patients in each group [18.8%]) and the frequency of previous percutaneous coronary intervention (TAVR, 51 patients [16.8%]; SAVR, 49 patients [16.1%]) were similar between the matched cohorts. Selected, more recent prostheses were used for 263 patients (86.5%) undergoing TAVR procedures and 150 patients (49.3%) undergoing SAVR procedures.

**Table 3.** Characteristics of Propensity Score–Matched Patients With Low Operative Risk Who Underwent TAVR or SAVR^a^

Characteristic, No. (%)	TAVR (n = 304)	SAVR (n = 304)	Standardized Difference (95% CI)	*P* Value
Age, y				
Mean (SD)	77.9 (6.0)	78.1 (4.8)	0.036 (−0.123 to 0.195)	.95
Median (IQR)	79.8 (75.4 to 82.0)	79.0 (74.4-82.1)		
Women	161 (53.0)	153 (50.3)	0.053 (−0.106 to 0.212)	.57
Body mass index^b^				
Mean (SD)	28.5 (5.1)	28.7 (4.9)	0.028 (−0.131 to 0.187)	.33
Median (IQR)	27.8 (24.7 to 31.9)	28.0 (24.8-31.6)		
Hemoglobin, g/dL				
Mean (SD)	13.1 (1.5)	13.0 (1.4)	0.04 (−0.12 to 0.20)	.60
Median (IQR)	13.2 (12.1 to 14.0)	13.0 (12.1-13.9)		
eGFR, mL/min/1.73 m^2^^c^				
Mean (SD)	76 (21)	76 (20)	0.01 (−0.15 to 0.17)	.95
Median (IQR)	73 (26)	74 (26)		
Diabetes	68 (22.4)	68 (22.4)	0 (−0.16 to 0.16)	>.99
Stroke	26 (8.6)	24 (7.9)	0.02 (−0.14 to 0.18)	.89
Transient ischemic attack	20 (6.6)	19 (6.3)	0.01 (−0.15 to 0.20)	>.99
Pulmonary disease	54 (17.8)	59 (19.4)	0.04 (−0.12 to 0.20)	.68
Extracardiac arteriopathy	39 (12.8)	42 (13.8)	0.03 (−0.13 to 0.19)	.81
LVEF ≤50%	41 (13.5)	40 (13.2)	0.01 (−0.15 to 0.17)	>.99
Atrial fibrillation	107 (35.2)	105 (34.5)	0.01 (−0.15 to 0.17)	.93
NYHA class 4	5 (1.6)	8 (2.6)	0.07 (−0.09 to 0.23)	.58
SPAP, mm Hg				
31-55	101 (33.2)	95 (31.3)	0.06 (−0.10 to 0.22)	.12
>55	15 (4.9)	18 (5.9)		
Moderate mitral regurgitation	20 (6.6)	19 (6.3)	0.04 (−0.12 to 0.20)	.80
Recent myocardial infarction	3 (1.0)	2 (0.7)	0.09 (−0.07 to 0.24)	>.99
Coronary artery disease	57 (18.8)	57 (18.8)	0 (−0.16 to 0.16)	>.99
Left main coronary stenosis	2 (0.7)	5 (1.6)	0.09 (−0.07 to 0.25)	.45
No. of diseased vessels				
Mean (SD)	0.2 (0.6)	0.3 (0.6)	0.03 (−0.13 to 0.19)	.92
Median (IQR)	0 (0)	0 (0)		
Prior PCI	51 (16.8)	49 (16.1)	0.02 (−0.14 to 0.18)	.91
Permanent pacemaker	21 (6.9)	15 (4.9)	0.08 (−0.08 to 0.24)	.41
Planned concomitant PCI or CABG	6 (2.0)	49 (16.1)	0.51 (0.35 to 0.67)	<.001
EuroSCORE II score, %				
Mean (SD)	2.6 (1.4)	2.5 (1.3)	0.04 (−0.12 to 0.20)	.65
Median (IQR)	2.2 (1.7 to 3.2)	2.2 (1.6 to 3.0)		
STS-PROM score, %				
Mean (SD)	2.1 (0.9)	2.1 (0.5)	0.03 (−0.14 to 0.18)	.82
Median (IQR)	2.2 (1.7 to 2.5)	2.2 (1.7 to 2.6)		

Abbreviations: CABG, coronary artery bypass grafting; eGFR, estimated glomerular filtration rate; EuroSCORE-II, updated European System for Cardiac Operative Risk Evaluation; IQR, interquartile range; LVEF, left ventricular ejection fraction; NYHA, New York Heart Association functional classification; PCI, percutaneous coronary intervention; SAVR, surgical aortic valve replacement; SPAP, systolic pulmonary artery pressure; STS-PROM, Society of Thoracic Surgeons Predicted Risk of Mortality; TAVR, transcatheter aortic valve replacement.

SI conversion factor: To convert hemoglobin to grams per liter, multiply by 10.

^a^ Clinical variables are according to the EuroSCORE II definition criteria.

^b^ Calculated as weight in kilograms divided by height in meters squared.

^c^ Calculated using the original Modification of Diet in Renal Disease equation.

Among the matched pairs, 30-day mortality included 4 patients (1.3%) after TAVR and 11 patients (3.6%) after SAVR (*P* = .12) (Table 4). Three-year survival was similar in the study cohorts (87.7% vs 85.7% for TAVR and SAVR, respectively; *P* = .45) (Figure).

**Table 4.** Early Outcomes in Propensity Score–Matched Low-Risk Patients Undergoing TAVR and SAVR

Outcome	No. (%)		*P* Value
	TAVR (n = 304)	SAVR (n = 304)	
Deaths at 30 d	4 (1.3)	11 (3.6)	.12
Conversion to cardiac surgery	3 (1.0)	NA	NA
Coronary ostium occlusion	2 (0.7)	2 (0.7)	>.99
Aortic dissection or rupture	2 (0.7)	5 (1.6)	.45
Major vascular complication	27 (8.9)	7 (2.3)	.001
Stroke	6 (2.0)	16 (5.3)	.12
RBC transfusion, units			
Mean (SD)	0.4 (1.2)	2.5 (2.9)	<.001
Median (IQR)	0 (0)	2.0 (0-4.0)	
RBC transfusion >4 units	7 (2.3)	46 (15.1)	<.001
Resternotomy for bleeding	1 (0.3)	18 (5.9)	<.001
E-CABG bleeding grades 2-3	7 (2.3)	51 (16.9)	<.001
KDIGO acute kidney injury grades 2-3	6 (2.0)	15 (5.0)	.12
Renal replacement therapy	1 (0.3)	5 (1.7)	.22
Paravalvular regurgitation			
Mild	46 (15.1)	17 (5.6)	<.001
Moderate	9 (3.0)	2 (0.7)	
Severe	0	0	
Atrial fibrillation	92 (30.3)	194 (63.4)	<.001
Permanent pacemaker	29 (9.5)	14 (4.6)	.03
Hospital stay, d			
Mean (SD)	4.1 (3.3)	7.9 (5.2)	<.001
Median (IQR)	3.0 (2.0-5.0)	7.0 (5.0-9.0)	

Abbreviations: E-CABG, European Coronary Artery Bypass Grafting registry; IQR, interquartile range; KDIGO, Kidney Disease: Improving Global Outcomes; NA, not applicable; RBC, red blood cells; SAVR, surgical aortic valve replacement; TAVR, transcatheter aortic valve replacement.

**Figure.**
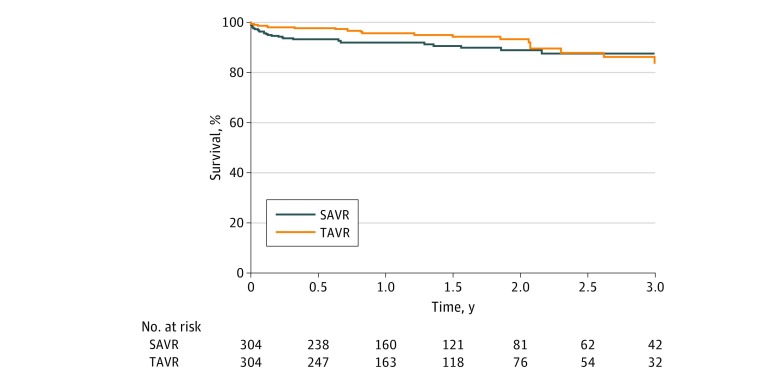
Kaplan-Meier Estimate of Survival in Low-Risk Patients With Aortic Stenosis Who Underwent Transcatheter (TAVR) or Surgical Aortic Valve Replacement (SAVR)

Patients who underwent TAVR had a shorter mean (SD) hospital stay (4.1 [3.2] days vs 7.9 [5.7] days; *P* < .001) and lower rates of atrial fibrillation (92 patients [30.3%] vs 194 patients [63.4%]; *P* < .001), resternotomy for bleeding (1 patient [0.3%] vs 18 patients [5.9%]; *P* < .001), and European Multicenter Study on Coronary Artery Bypass Grafting bleeding grades 2 or 3 (7 patients [2.3%] vs 51 patients [16.9%]; *P* < .001) compared with patients who underwent SAVR (Table 4). Two patients in each cohort had coronary ostium occlusion, and conversion to cardiac surgical operations was necessary for 3 patients in the TAVR cohort. Major vascular complications were more frequent in the TAVR cohort than the SAVR cohort (27 patients [8.9%] vs 7 patients [2.3%]; *P* = .001), and patients who received TAVR experienced higher rates of paravalvular regurgitation with SAVR (mild regurgitation: TAVR, 46 patients [15.1%]; SAVR, 17 patients [5.6%]; moderate regurgitation: TAVR, 9 patients [3.0%]; SAVR, 2 patients [0.7%]; *P* < .001).

No significant difference was observed in the rates of stroke between the cohorts. There was a higher rate of acute kidney injury in the SAVR cohort (15 patients [5.0%]) compared with the TAVR cohort (6 patients [2.0%]), but the difference was not significant (*P* = .12). Permanent pacemaker implantation was needed more often among patients who underwent TAVR than those who underwent SAVR (29 patients [9.5%] vs 14 patients [4.6%]; *P* = .03).

The interaction test for type of procedure and CAD was statistically significant and demonstrated unfavorable intermediate survival in patients with CAD who underwent SAVR (eTable in the Supplement). Interaction tests found that survival in the TAVR cohort was similar to that of the SAVR cohort for patients younger than vs older than 80 years (*P *for interaction = .23) and for patients who received selected valve prostheses vs those who did not (*P *for interaction = .26) (eTable in the Supplement).

## Discussion

This nationwide study represents one of the largest studies of low-risk patients who underwent TAVR or SAVR, to our knowledge. We found that short-term and midterm mortality in low-risk patients was low and similar after TAVR or SAVR, TAVR was associated with shorter hospital stays and a favorable safety profile in terms of major perioperative bleeding, and SAVR was associated with lower rates of severe vascular complication, paravalvular regurgitation, and need of permanent pacemaker.

This study represents a nationwide practice demonstrating that from January 1, 2008, to November 30, 2017, 325 of 2841 patients (11.4%) with AS and low operative risk were treated with TAVR in Finland. Slightly lower proportions of low-risk patients during a similar time frame was reported by Schymik et al.^20^ An STS-PROM score less than 3.0% was selected as the cutoff value for low-risk patients, which is supported by earlier studies.^10,21^ Patients who have generally a higher operative risk, such as patients older than 85 years with severe frailty and a recent acute heart failure episode, were excluded, leaving a small proportion of patients with comorbidities.

Initially, TAVR was indicated only for inoperable or high–operative risk patients, but accepted clinical practice has expanded to perform TAVR in patients at lower operative risk^7,8,22^ and requires more data on long-term outcomes in these populations. To our knowledge, the longest follow-up data on similar survival after TAVR or SAVR extend 5 years for high-risk patients,^1,3^ while data on intermediate survival among low-risk patients are limited.^5,6,10^ Despite the short-term results documented with TAVR compared with SAVR in low-risk patients from two 2019 randomized clinical trials,^7,8^ there are limited data on the midterm and long-term outcomes after TAVR in low-risk patients. Our data suggest that similar and excellent survival can be expected 3 years after TAVR (85.7%) and SAVR (87.7%) in patients deemed to have low operative risk. Indeed, the 3-year survival of patients who underwent TAVR was higher than reported in a low-risk series from the 2016 Observational Study of Effectiveness of SAVR-TAVI Procedures for Severe Aortic Stenosis Treatment (OBSERVANT),^12^ in which the TAVR cohort had inferior midterm survival compared with the SAVR cohort. However, OBSERVANT gathered data from 2010 to 2012 with limited operator experience and using only second-generation TAVR devices.^12^ The higher rate of significant paravalvular regurgitation might have been negatively associated with long-term outcomes observed in OBSERVANT, a phenomenon well documented in other studies.^3,4,23^ Indications for TAVR expanded to include low-risk patients only in recent years; therefore, 86.5% of low-risk patients in the FinnValve registry who underwent TAVR received third-generation TAVR devices and were treated by more experienced operators. Minimal-risk patients in the TAVIK registry^20^ had a lower 2-year survival after TAVR (90.9%) compared with SAVR (95.7%; *P* = .001), a difference that was likely associated with older age in the TAVR cohort. The NOTION trial randomized mainly low-risk patients (mean STS-PROM score, 3%; mean EuroSCORE II score, 2%) to receive TAVR or SAVR and demonstrated similar survival rates at 6 years (57.5% vs 62.3%), despite a 15% rate of moderate aortic regurgitation in the TAVR arm.^9,10^

To our knowledge, this study is one of few providing 3-year follow-up survival after TAVR and SAVR in low-risk patients with AS. Numerically higher 30-day mortality was observed after SAVR in the overall (50 of 2516 patients [2.0%]) and matched (11 of 304 patients [3.6%]) populations. It is worth noting that 30-day mortality after TAVR was lower than predicted by the STS-PROM and EuroSCORE II scores in the overall (4 of 325 patients [1.2%]) and matched (4 of 304 patients [1.3%]) populations. The same was not observed in the SAVR cohort. These findings are similar to those of previous studies in low-risk populations, which reported in-hospital 30-day mortality of 1% to 2% in both TAVR and SAVR populations.^11,20,24^ A 2018 study by Waksman et al^11^ found no mortality at 30 days among low-risk patients who underwent TAVR. However, the study by Waksman et al^11^ consisted of patients much younger than other studies. Overall, current data suggest that very low operative mortality can be expected when treating low-risk patients older than 70 years with TAVR or SAVR techniques. Indeed, in the 2019 Placement of Aortic Transcatheter Valves (PARTNER) 3 trial,^8^ 30-day mortality was 0.5% after TAVR and 1.3% after SAVR, and in the Evolut Low Risk trial,^7^ 30-day mortality was 0.4% after TAVR and 1.1% after SAVR.

Nearly half of patients treated for AS have concomitant CAD.^25^ In our study of selected low-risk patients, less than 1 of 5 patients had significant CAD. The prevalence of CAD and history of percutaneous coronary intervention were comparable in the study cohorts. However, only 15% of patients with CAD undergoing TAVR underwent any planned coronary revascularization, while 93% of patients with CAD undergoing SAVR underwent a concomitant revascularization. This reflects the contemporary practice of accomplishing revascularization concomitantly with SAVR if CAD is detected by a preoperative coronary angiography, as supported by the 2017 European Society of Cardiology and European Association for Cardio-Thoracic Surgery guidelines^26^ and the 2014 American Heart Association and American College of Cardiology guideline.^27^ Patients undergoing combined SAVR and coronary surgery procedures are potentially exposed to a higher risk of early mortality, most likely owing to CAD itself as well as to prolonged intraoperative myocardial ischemia. In these patients, cardiac surgeons are prone to perform coronary artery bypass grafting and administer antegrade cardioplegia through vein grafts to avoid suboptimal myocardial protection during prolonged cardiopulmonary bypass and to decrease the risk of possible ischemic complications early after SAVR. The risk of myocardial ischemia is lower after TAVR because of the minimally invasive nature of this treatment, which does not require the use of cardiac arrest during cardiopulmonary bypass. It is worth noting that leaving CAD untreated during SAVR impairs long-term survival regardless of the severity of CAD.^28^ In the present study, we observed that concomitant CAD was associated with worse outcomes in patients undergoing SAVR (eTable in the Supplement). It is also possible that increased operative mortality in the SAVR cohort was partly owing to a higher proportion of combined procedures, although the revascularization rate was performed in only 16% of the matched patients in the SAVR cohort. The association of concomitant CAD with long-term mortality after TAVR is controversial,^25,29^ and the indication of percutaneous coronary intervention prior to or during TAVR is commonly discussed before valve intervention. The severity of CAD and success of revascularization may have a role in survival after TAVR, and these components have to be considered during decision making.^30^ Interpreting the results of studies about AS and coexisting CAD becomes more difficult when considering the hemodynamic severity of CAD,^31,32^ and hopefully the ongoing Percutaneous Coronary Intervention Prior to Transcatheter Aortic Valve Implantation ACTIVATION (ISRCTN75836930) and NOTION-3 (NCT03058627) trials will provide conclusive data on the potential benefits of percutaneous coronary intervention during TAVR.

The distribution of periprocedural complications in our study was typical compared with another low-risk study by Witberg et al.^33^ Bleeding and atrial fibrillation were less frequent in the TAVR cohort, and less vascular complications and new permanent pacemaker implantations were observed in the SAVR cohort. Higher rates of acute kidney injury and stroke were observed after SAVR, but severe intraprocedural complications were infrequent. Major bleeding events were significantly higher in the SAVR cohort than the TAVR cohort.

### Limitations

The main limitation of this study is its retrospective nature. Second, the definition of low risk was based on a cutoff value of 3% for operative mortality as estimated by the STS-PROM scoring system and by excluding patients deemed at increased risk because of significant comorbidities. Despite these inclusion criteria, it is possible that some patients included or excluded from this analysis were incorrectly classified. Third, comparative analysis of the study cohorts was based on propensity score matching, and its results are potentially biased by unmeasured confounders despite well-balanced covariates. Fourth, the relatively small size of the matched study cohorts may affect the reliability of these results. Fifth, the limited length of follow-up of patients with low operative risk prevented more conclusive results on the durability of TAVR in this patient population.

## Conclusions

This nationwide registry analysis found that TAVR using mostly third-generation devices achieved similar early and intermediate survival compared with SAVR in low-risk patients. Before extending the use of TAVR to low-risk patients, further studies are needed to assess the long-term durability of transcatheter aortic valve prostheses.

## Notes

Supplement. **eFigure.** Study Flowchart **eTable.** Interaction Tests for Intermediate Mortality in Subgroups of Low-Risk Patients Undergoing Transcatheter or Surgical Aortic Valve Replacement
