# Errors in Key Points and Results Sections

**DOI:** 10.1001/jamanetworkopen.2019.8352

**Published:** 2019-07-12

In the Original Investigation titled “Comparison of Outcomes After Transcatheter Aortic Valve Replacement vs Surgical Aortic Valve Replacement Among Patients With Aortic Stenosis at Low Operative Risk,”^1^ published June 14, 2019, there was a typographical error in the Key Points section. In the Results section, in the second paragraph of the Characteristics and Outcomes of the Propensity Score–Matched Cohorts subsection, the 3-year survival rates were transposed and should have read “Three-year survival was similar in the study cohorts (85.7% vs 87.7% for TAVR and SAVR, respectively; *P* = .45).” This article has been corrected.^1^
