# Supplementary material for: Comparison of Outcomes After Transcatheter Aortic Valve Replacement vs Surgical Aortic Valve Replacement Among Patients With Aortic Stenosis at Low Operative Risk
Source: JAMA Netw Open. 2019 Jun 14;2(6):e195742. doi: 10.1001/jamanetworkopen.2019.5742 (PMC6575142; doi:10.1001/jamanetworkopen.2019.5742)
Supplement: Supplement. — eFigure. Study Flowchart eTable. Interaction Tests for Intermediate Mortality in Subgroups of Low-Risk Patients Undergoing Transcatheter or Surgical Aortic Valve Replacement [file jamanetwopen-2-e195742-s001.pdf]

Supplementary Online Content

Virtanen MPO, Eskola M, Jalava MP, et al. Comparison of outcomes after transcatheter aortic valve replacement vs surgical aortic valve replacement among patients with aortic stenosis at low operative risk. *JAMA Netw Open*. 2019;2(6):e195742. doi:10.1001/jamanetworkopen.2019.5742

eFigure. Study Flowchart

eTable. Interaction Tests for Intermediate Mortality in Subgroups of Low-Risk Patients Undergoing Transcatheter or Surgical Aortic Valve Replacement

This supplementary material has been provided by the authors to give readers additional information about their work.

© 2019 Virtanen MPO et al. *JAMA Network Open.*

eFigure. Study Flowchart

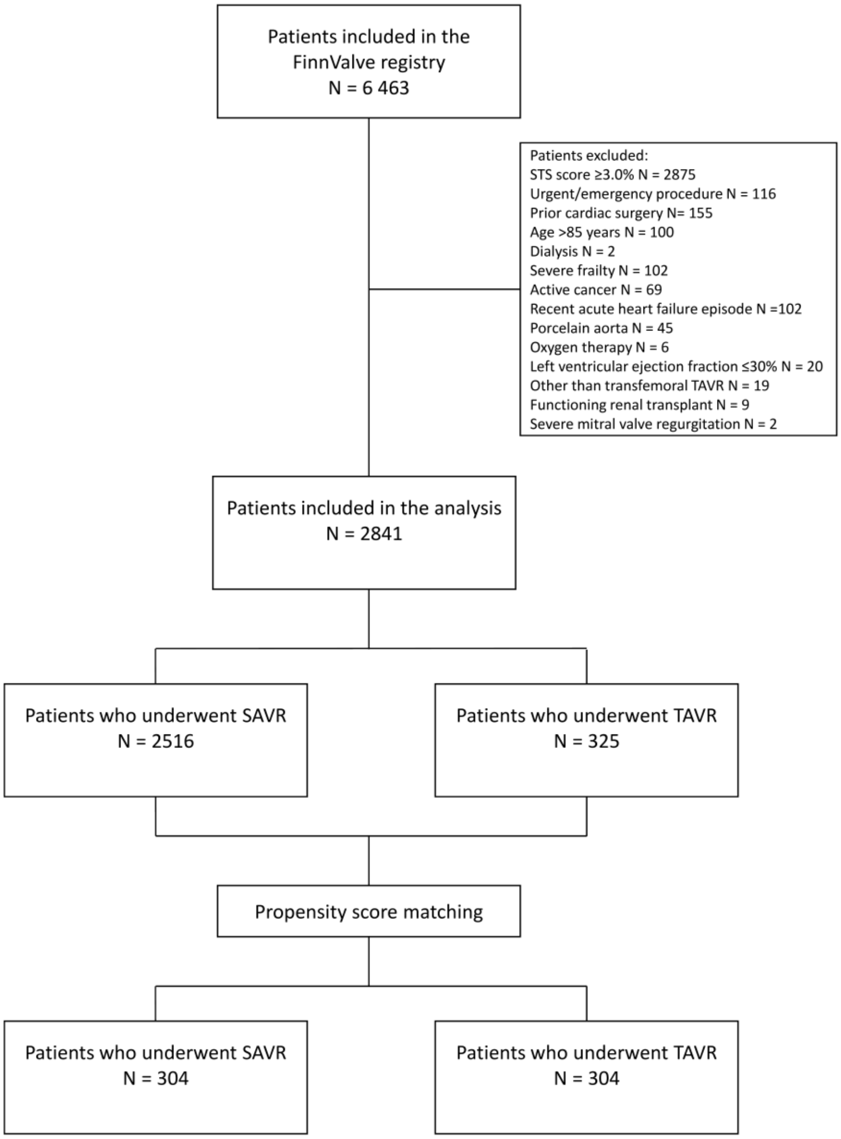

© 2019 Virtanen MPO et al. *JAMA Network Open.*

eTable. Interaction Tests for Intermediate Mortality in Subgroups of Low-Risk Patients Undergoing Transcatheter or Surgical Aortic Valve Replacement

	Covariates	SAVR No. of pts	TAVR No. of pts	Hazard ratio	95% confidence interval		Interaction p-value
SAVR vs. TAVR	Overall	304	304	1.393	0.784	2.472	
SAVR vs. TAVR	Coronary artery disease	57	57	3.242	0.736	14.273	0.078
SAVR vs. TAVR	No coronary artery disease	247	247	0.753	0.385	1.473	
SAVR vs. TAVR	Age ≥80 years	143	142	1.502	0.608	3.713	0.234
SAVR vs. TAVR	Age <80 years	161	162	0.64	0.217	1.887	
SAVR vs. TAVR	Other than selected prostheses	154	41	0.492	0.135	1.783	0.264
SAVR vs. TAVR	Selected prostheses	150	263	1.138	0.559	2.316	

SAVR, surgical aortic valve replacement; TAVR, transcatheter aortic valve replacement.

© 2019 Virtanen MPO et al. *JAMA Network Open.*
